# Response of *Escherichia coli* to Acid Stress: Mechanisms and Applications—A Narrative Review

**DOI:** 10.3390/microorganisms12091774

**Published:** 2024-08-28

**Authors:** Zepeng Li, Zhaosong Huang, Pengfei Gu

**Affiliations:** School of Biological Science and Technology, University of Jinan, Jinan 250022, China; 13953161475@163.com (Z.L.); bio_huangzs@ujn.edu.cn (Z.H.)

**Keywords:** *Escherichia coli*, acid-tolerance mechanism, cell membrane protection, macromolecular repair, industrial applications

## Abstract

Change in pH in growth conditions is the primary stress for most neutralophilic bacteria, including model microorganism *Escherichia coli*. However, different survival capacities under acid stress in different bacteria are ubiquitous. Research on different acid-tolerance mechanisms in microorganisms is important for the field of combating harmful gut bacteria and promoting fermentation performance of industrial strains. Therefore, this study aimed to carry out a narrative review of acid-stress response mechanism of *E. coli* discovered so far, including six AR systems, cell membrane protection, and macromolecular repair. In addition, the application of acid-tolerant *E. coli* in industry was illustrated, such as production of industrial organic acid and developing bioprocessing for industrial wastes. Identifying these aspects will open the opportunity for discussing development aspects for subsequent research of acid-tolerant mechanisms and application in *E. coli*.

## 1. Introduction

As a model microorganism, *E. coli* possesses the advantages of clear genetic background, convenient metabolic engineering tools, and fast growth in cheap media. Accordingly, *E. coli* is widely applied as a microbial cell factory for the production of various valuable chemicals [1,2]. On the other hand, *E. coli* is closely related to human daily life, which is accounting for about 1% of the intestinal bacteria in humans and animals [3,4]. As a class of intestinal strains, it can mainly divide into pathogenic *E. coli* and non-pathogenic *E. coli* [5,6]. The pathogenic *E. coli* exhibited a worldwide distribution leading to epidemic infections and it represents a predominant group of infecting bacteria [7,8]. In addition, this pathogenic *E. coli* is often able to enter into the intestinal tract through the human stomach (pH = 1.5~3.5) and poses a threat to human health. Accordingly, analysis of the acid-resistant mechanism is useful for dealing with pathogenic *E. coli*. However, most *E. coli* strains are harmless, which not only produces vitamin K for humans in the intestinal tract, but also inhibits the growth and reproduction of pathogenic bacteria.

In the natural environment, bacteria strains usually encounter complex and uncomfortable conditions, such as acid, alkali, high or low temperature, and antibiotics. As microorganisms can secrete organic acid as by-products, the acid environment becomes one of the most typical uncomfortable environmental conditions that bacteria regularly endure [9]. Change in pH in growth conditions is the primary stress for most neutralophilic bacteria. However, different survival capacities under acid stress in different bacteria are ubiquitous [10,11,12]. For example, the intestinal microbiology must encounter a wide range of pH values before colonization into the human gut, including the stomach with a pH ranging from 1.5~3.5, duodenum with a pH rising from 4~7, jejunum with a pH ranging from 7~9, and cecum with a pH dropping to 5~6 [13,14]. It was reported that several *E. coli* strains have evolved to grow over a relatively wide range of intestinal pH ranging from 4.5~9.0 [15,16], and can even survive for several hours at pH = 2 but cannot grow [17].

In addition, maintaining relatively stable pH during microbial fermentation of valuable chemicals is curtailed for the final titer, yield, and productivity of target products. When an excess carbon source such as glucose was supplemented into the medium, organic acid such as formic acid, acetic acid, and lactic acid could be generated due to the imbalance of the glycolytic pathway and TCA cycle. As a result, the pH of the medium usually dropped below 5.0, which severely inhibited the normal growth of *E. coli* used for microbial production. Recently, it has been found that organic acid titers of about 50 g/L with pKa 3–5 will lower the environmental pH to about 2.0 in the absence of alkali intervention, and the cell membrane structure and intracellular homeostasis will be destroyed, leading to cell death and severely affecting productivity [18]. To overcome this inhibiting effect, an alkaline substance such as ammonium hydroxide, sodium hydroxide, sodium carbonate, or sodium bicarbonate should be added which increase the total cost of fermentation. Therefore, development of an acid-resistant *E. coli* could save the cost of alkaline substance and facilitate the fermentation process with a relatively wide pH range.

As mentioned above, the study of acid-tolerance mechanisms of different microorganisms is important to combat harmful gut bacteria and to reduce the total cost of industrial fermentation. Microorganisms have developed various complex mechanisms to survive and adapt to acid stress at both physiological and molecular levels. In addition, several novel approaches have been applied to investigate the acid-tolerance mechanisms of different microorganisms [9]. Although the research on acid resistance of a range of microorganisms has achieved a stage-by-stage victory, it still needs to be explored in depth in the face of certain extreme acid environments and growing social demands. Here, we reviewed the acid-stress response mechanism of *E. coli* in detail and illustrated the present and potential application of acid-tolerant *E. coli* in industry. In addition, the development prospects and challenges of acid-tolerant *E. coli* were discussed.

## 2. Methods

This narrative review was carried out by three steps including searching relevant articles, reviewing abstracts and full texts, and analyzing results as previously reported [19]. The PubMed, Scopus, Science Direct, and Web of Science databases were searched to collect relevant studies, according to the topic of the review. The keyword “acid-tolerant *Escherichia coli*” was employed combined with other terms such as “acid-resistance systems”, “cell membrane protection”, and “macromolecular repair”. After the complete search, the abstracts were read to ensure that they address the topic of interest. All duplicates were removed, and the abstracts of the remaining articles were reviewed to ensure that they address the review inclusion criteria. The eligible criteria were analyzed in at least one of the two dimensions (acid-tolerance mechanism of *E. coli* or industrial application of acid-tolerance *E. coli*). Therefore, studies focusing on the acid-tolerance mechanism of *E. coli* or industrial application of acid-tolerance *E. coli* were summarized and synthesized to integrate a narrative review. Since it is a narrative review, it was unnecessary to document the literature search on specific platforms.

## 3. Mechanism of Different Acid-Resistance Systems in *E. coli*

**Figure 1:**
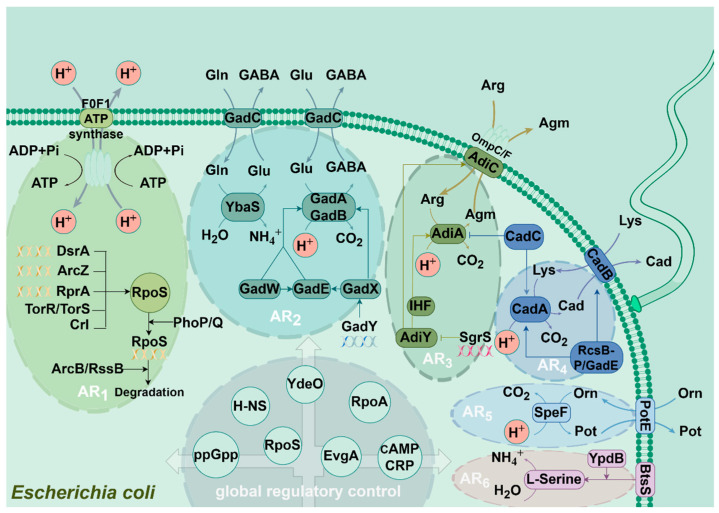
Schematic diagram of the acid-resistance system of *Escherichia coli* and its regulatory network.

Due to the complexity of the human gut, E. coli strains have developed various acid-stress response systems, including the acid-resistance (AR) system for extreme acid-stress responses and the acid-tolerance (ATR) system for mild and moderate acid-stress responses [20,21]. Until now, six AR systems have been identified and analyzed, which have been named from AR_1_ to AR_6_ (Figure 1).

### 3.1. AR_1_

AR_1_ is an oxidized AR system, which is also known as the glucose-inhibitory system and exhibited acid-induced in quiescent cells. Its expression requires the activation of RpoS and the cAMP receptor protein (CRP) [22,23]. RpoS plays a key role in the AR_1_ system. Once RopS is inactivated, the AR_1_ system will directly lose its acid-tolerance function. In *E. coli*, the expression of RpoS is regulated by a variety of factors. For example, small sRNAs such as RprA, DsrA, and ArcZ can activate the expression of RpoS [24], PhoP/PhoQ can indirectly regulate the transcription of RpoS, ArcB/RssB can indirectly inhibit the degradation of RpoS, TorR/TorS system can increase and stabilize the expression of RpoS [24], and transcriptional regulator Crl can control RpoS expression [25]. In addition, CRP is one of the seven global regulators in *E. coli*, responsible for regulating about 490 genes [26]. CRP is composed of a cAMP-binding N-terminal structural domain, a DNA-binding C-terminal structural region, and a linker hinge region [27], of which the function needs to be activated by cAMP. The cAMP is a second messenger produced by most of the organisms when it receives environmental stimuli. cAMP can bind to CRP when its concentration exceeds the threshold value, thus activating the expression of the AR_1_ system. On the other hand, a high concentration of glucose will inhibit the adenylate cyclase activity, which will interfere with the binding of cAMP to CRP in turn. As a result, the R_1_ system will be out of operation. This phenomenon also explains why the AR_1_ system can also be called the glucose-inhibitory system. However, its structural components and detailed acid-resistant mechanism are still unknown.

It was reported that F_0_F_1_-ATPase is also associated with the AR_1_ system. The F_0_F_1_-ATPase consists of a catalytic part F_1_ and a proton channel F_0_ that plays a central role in bioenergetic transduction. F_0_F_1_-ATPase is able to be activated by glutamate and is inhibited by glucose. Although F_0_F_1_-ATPase is bifunctional and able to participate in ATP synthesis and hydrolysis, F_0_F_1_-ATPase rapidly shifts its mechanism to consume intracellular H^+^ by hydrolyzing ATP to maintain intracellular homeostasis when *E. coli* is subjected to acid stress [22,28].

### 3.2. AR_2_

AR_2_ is a glutamate-dependent acid-tolerance system, which is also known as the Gad system. AR_2_ was found to be the most effective mechanism of acid resistance in *E. coli*. With the help of AR_2_, *E. coli* can maintain intracellular pH homeostasis by consuming glutamate at pH 2.5, thus enabling *E. coli* to survive at extreme acidic environments for several hours without growth. The glutamate-dependent AR system is regulated by two isoforms of glutamate decarboxylase (GadA and GadB) and the anti-transporter protein GadC [29]. It was found that the Gad system can decarboxylate intracellular glutamate into γ-aminobutyric acid (GABA) and carbon dioxide via GadA and GadB combined with intracellular H^+^ consuming. And then, the generated GABA can be transported out of the cell by a specific anti-transporter system GadC in exchange for a new glutamate substrate. This process can prevent the internal pH from dropping to lethal levels [30,31].

It was found that deletion of all three key genes, *gadA*, *gadB,* and *gadC* can significantly affect bacterial survival at pH 2~3. Moreover, the presence of one decarboxylase, whether GadA or GadB, is sufficient for *E. coli* survival at pH 2.5. However, survival at pH 2 requires both glutamic acid decarboxylases GadA and GadB to function simultaneously [18]. In addition, GadA and GadB are differentially expressed at the pH 5~7 [32,33,34].

In addition to glutamine as a substrate, the Gad system is also regulated by the glutaminase YbaS and the membrane transporter protein GadC when glutamine concentrations are high and the ambient pH is below 6.0. When glutaminase YbaS is activated by an acidic stimulus, it converts absorbed glutamine to glutamate and releases gaseous ammonia. The free ammonia can neutralize intracellular protons, thus maintaining cellular homeostasis from acid stress [35,36]. Moreover, the enzymatic reaction of glutamine and the decarboxylation reaction of glutamate can function simultaneously without interfering with each other, which can consume two H^+^ at a time, greatly enhancing the acid resistance of *E. coli*.

The Gad system can maintain its normal intracellular levels by cycling through the transporter-decarboxylator-anti-transporter cycle, which allows for the expulsion of incoming protons and the replacement of reaction products with extracellular substrates. Although the acid-tolerance mechanism of the Gad system appears relatively simple, it is influenced by a variety of regulatory genes. Apart from GadA, GadB, and GadC, more than 20 other regulatory genes have been involved. Among the regulatory genes, GadE, GadX, and GadW play a major role. GadE, located in the upstream of GadA, is part of the LuxR regulatory proteins family [37], which is a central transcriptional activator of GadABC [37,38]. The expression of GadE can be directly or indirectly regulated by GadX and GadW, and the degradation of GadX and GadW can be promoted by overexpression of RpoS. In addition, the sRNA of GadY can bind to the region of GadX and lead to GadX accumulation, which in turn activates the expression of Gad system genes. It was reported that other regulatory proteins, such as YhiM, SdiA, YfdW, YfdU, EvgS, EvgA, YdeO, TrmE, RcsB, Crp, TorR, PhoP, H-NS, HdfR, RcsB-P, and Gad also participate in the expression of the Gad system’s function under different acidic conditions regulate [39,40,41,42,43].

### 3.3. AR_3_

AR_3_ is an arginine-dependent acid-tolerance system, also known as the Adi system, which is similar to the glutamate-dependent acid-tolerance system. With the help of the Adi system, the effect of acid stress for microorganisms can be effectively mitigated at pH 2.5. This system consists of the acid-induced arginine decarboxylase AdiA and the anti-transporter protein AdiC [44,45]. Similar to the Gad system, the Adi system can decarboxylate transferred arginine and consume intracellular H^+^ through a decarboxylation reaction in the meantime. Subsequently, the decarboxylation product guanidinium butylamine is excreted in exchange for a new arginine and begins a transporter-decarboxylator-anti-transporter cycle.

The Adi system is also regulated by a variety of regulatory proteins like the Gad system. As an anti-transporter protein, the function of AdiC is similar to GadC in the Gad system, and is located upstream of the arginine decarboxylase AdiA [44,46,47]. In addition, it was found that regulatory factors such as CadC, OmpC/OmpF, AdiY, RpoA, and SgrS can promote or inhibit the Adi system, respectively [40,48,49].

### 3.4. AR_4_

AR_4_ is a lysine-dependent acid-tolerant system, also known as the Cad system. This system consists of a lysine decarboxylase CadA and an anti-transporter protein CadB, which normally functions in a mildly acidic environment around pH 5.8. It has been found that the lysine-dependent acid-tolerance system has low activity in *E. coli* but shows high activity in *Salmonella* sp. The CadA of the Cad system can decarboxylate intracellular lysine into glutamine and carbon dioxide, which consumes intracellular H^+^. And then, CadB is responsible for pumping glutamine out of cells and exchanging it for a new substrate, which can keep the intracellular pH in a normal range [50].

The Cad system is mainly regulated by the ToxR family CadC, a key protein for transcriptional activation of *cadBA* [51]. Previously, the lysine-specific permease LysP was found to regulate the expression of *cadA*, which binds to CadC and activates the expression of *cadBA* at the same time, but this regulation requires both low pH and high lysine concentration [51,52,53]. In addition, the Cad system was found to be regulated by the RcsB-P/GadE regulatory complex and H-NS. The former is essential for the lysine-dependent acid-tolerance system and directly regulates CadA and CadB, while the latter directly controls expression of *cadC*, a regulator of this system [40].

### 3.5. AR_5_

AR_5_ is an ornithine-dependent acid-tolerant system, also known as the Orn system, which can be activated at pH 5.0 anaerobically and maintains intracellular homeostasis by depleting ornithine. AR_5_ consists of an ornithine decarboxylase SpeF and an anti-transporter protein PotE [54]. This system decarboxylates ornithine into putrescine and carbon dioxide via ornithine decarboxylase SpeF and consumes intracellular H^+^, while the anti-transporter protein PotE is responsible for exchanging putrescine with extracellular ornithine [55]. However, this system is less efficient than the glutamate- or arginine-dependent acid-tolerant system in *E. coli*.

### 3.6. AR_6_

Recently, a novel AR mechanism named AR_6_ in pathogenic *E. coli* was identified which is controlled through a non-classical signaling system, BtsS-YpdB. *E*. *coli* contains two LytS/LytTR-type his-tidine kinase/response regulator systems, BtsS/BtsR (formerly YehU/YehT) and YpdA/YpdB, which have been identified as pyruvate-responsive two-component systems. We found that BtsS/BtsR is the predominant LytS/LytTR-type two-component system among γ-proteobacteria, whereas YpdA/YpdB primarily appears in a supplementary role. In *E. coli*, the functional link between the two systems provides for positive feedback from YpdA/YhjX to YjiY (a CstA-like transport protein) transcription, while yhjX (a putative antiporter of the MF superfamily) is negatively regulated by BtsS/YjiY activity [56,57]. This system can neutralize bacterial cytoplasmic pH by L-serine deamination. However, the detailed acid-resistance mechanism of AR_6_ is not clear and needs to be further investigated in the future [56].

## 4. Cell Membrane Protection

**Figure 2:**
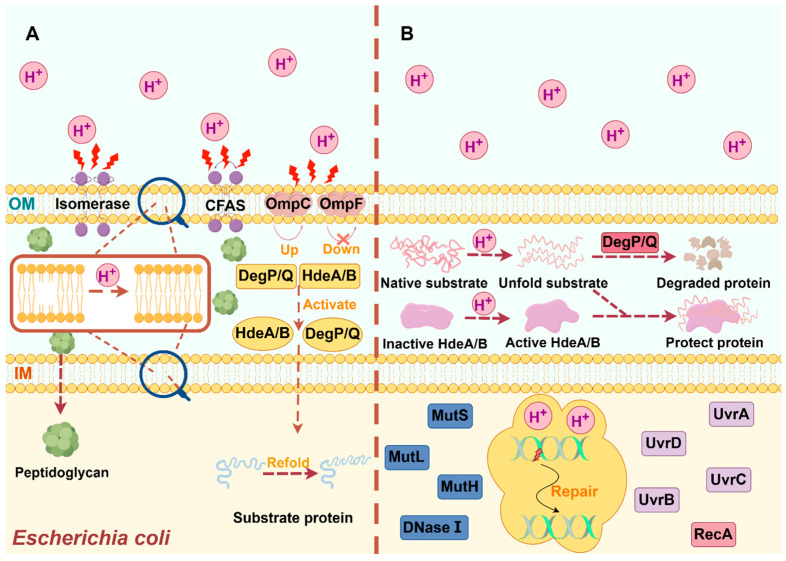
Protection and repair mechanism of acid-resistant cells in *Escherichia coli*. (**A**) Protection of cell membrane. (**B**) Repair of macromolecule.

Apart from the AR system, the cell membrane also plays an integral role in the acid-tolerance mechanism of *E. coli* (Figure 2A). In *E. coli*, the cell membrane contains both outer and inner membranes, but the inner membrane plays a more important role for acid tolerance. The *E. coli* cell membrane can inhibit proton influx and maintain membrane integrity and stability. The membrane components, including lipids, lipopolysaccharide (LPS), pore proteins, chaperonins, and periplasmic peptidoglycan are important for acid resistance of *E. coli* [11]. Membrane lipids can enhance acid resistance by altering the ratio of saturated/unsaturated fatty acids via isomerase or by converting unsaturated fatty acids to more stable cyclopropane fatty acids via cyclopropane fatty acid synthetase [58]. In addition, LPS is an amphiphilic membrane component consisting of hydrophilic polysaccharides (O-antigen and core oligosaccharide) and hydrophobic lipids containing 3-deoxy-alpha-D-manno-oct-2-ulopyranosonic acid (Kdo). It has been found that modulating the charge state of LPS could improve cellular acid resistance. LPS can interact with divalent cations present in the environment and neutralize phosphorylation of phospholipids and Kdo sugars, thereby reducing a high negative charge. In addition, changes in LPS composition also enhanced acid resistance by increasing PagP expression through PhoP/Q [59,60].

In addition, pore proteins also play a very important role in acid tolerance of *E. coli*, usually combined with other acid-tolerance mechanisms. The OmpC and OmpF proteins are employed as input channels for arginine and lysine, respectively, under acidic conditions. By regulating these pore protein channels, the inputs of arginine and lysine are changed, ultimately affecting the AR_3_ and AR_4_ system in turn and controlling the entry and exit of protons in the meantime [48,61]. It was found that overexpression of OmpC and down-regulation of OmpF contributed to acid tolerance of *E. coli*, but further investigation is still needed to explore the detailed mechanism [62].

In *E. coli*, chaperonins and peptidoglycans are responsible for protecting key proteins from acid damage. Chaperone proteins can assist the folding/unfolding and assembly/disassembly of HdeA/HdeB and DegP/DegQ. HdeA and HdeB are two structurally related periplasmic chaperones, and both of them can fold into an inactive dimer when *E. coli* is in a mild or moderate acidic environment. It was found that HdeA works mainly under extreme acid stress (pH 1~3), while HdeB works mainly under mild or moderate acid stress (pH 3~5) [63]. In extremel acidic environments, they are converted to a highly plastic monomeric conformation, which promotes the folding of other acid-resistant-related molecules and prevents macromolecule aggregation which results from high intracellular concentrations of hydrogen and chloride ions without the need for ATP [64]. In contrast, DegP/DegQ promotes the degradation of misfolded proteins. It was found that DegP would completely lose its protease activity under acid stress, but regain some of its activity after neutralization [65,66].

On the other hand, periplasmic peptidoglycan is another important component for maintaining structural stability when *E. coli* is under mild or moderate acid stress. Even when damaged by acid stress, *E. coli* can still remodel peptidoglycan and maintain the shape of the cell by synthesizing and hydrolyzing peptidoglycanases [59,67]. It was also found that D-alanyl-D-alanine carboxypeptidase (DD-CPase) was very active under acidic conditions, especially PBP6b in DD-CPase, which was able to transform peptidoglycan into pentapeptide by transpeptidases in an acidic environment and maintain cell shape effectively under an acidic environment [68]. Peptidoglycan, a major component of periplasm, plays an integral role in the acid resistance of *E. coli* [69].

## 5. Macromolecular Repair

The structure and activity of intracellular biomolecules such as proteins and nucleic acids are probably changed when *E. coli* is subjected to acid stress. With acidification of cytoplasm, bases are protonated resulting in the breakage of glycosyl bonds and depurination and depyrimidination of DNA in turn. Furthermore, protons will react with amino acid residues of proteins, leading to protein aggregation, unfolding, and even denaturation [70] (Figure 2B). As a result, DNA and chaperone repair systems are vital for ensuring cell survival after acid stress. In response to DNA damage, *E. coli* has evolved a variety of DNA repair mechanisms, including nucleotide excision repair (NER), mismatch repair (MMR), base excision repair (BER), and RecA protein repair pathways. Nucleotide excision repair (NER) can remove helix-distorting lesions from DNA to ensure genomic integrity. NER, exclusively mediated by DNA transposase Mfd, has traditionally been divided into major and minor sub-pathways, called genome-wide nucleotide excision repair (GGR) and transcription-coupled nucleotide excision repair (TCR), respectively. In *E. coli*, nucleic acid endonucleases UvrABCD are required to recognize and cleave to both sides of the damaged nucleotide [71]. Mismatch repair (MMR) is a highly conserved DNA repair system that repairs base–base mismatches and insertion/deletion loops that often occur during DNA replication. The homodimer MutS initially recognizes DNA mismatches and then recruits the homodimer MutL and related proteins downstream, which interact with each other to achieve DNA mismatch repair [72]. Base excision repair (BER), mainly consisting of lesion-specific DNA glycosylases and adenine/pyrimidine (AP) nucleic acid endonucleases, is one of the most important DNA repair mechanisms for repairing mononuclear base damage produced by reactive oxygen and nitrogen species [73]. In addition, RecA is essential for double-strand break repair (DSBR), natural transformation, and SOS responses in *E. coli*. The RecA protein can form nucleoprotein filaments by encapsulating single-stranded DNA [74], which is regulated by *recN* and works with the RecBCD complex to repair DNA [66,74].

## 6. Potential Application and Development of Acid-Resistant *E. coli* in Industry

As reviewed above, *E. coli* can sense and respond to acid stress in the environment through a variety of pathways, such as consuming protons, cell membrane protection, and macromolecular repair. Development of acid-resistant *E. coli* will provide a broad prospect for future industrial applications and environmental remediation, such as microbial production of industrial organic acid and biofuel, and developing bioprocessing for industrial wastes [75]. Traditionallly, *E. coli* is not the preferred host for organic acid production due to its relatively low tolerance for the acid environment. As a result, developing acid-resistant *E. coli* is advantageous for its organic acid production. In addition, maintaining the relatively stable level of pH during microbial fermentation of valuable chemicals is curtailed for the final titer, yield, and productivity in *E. coli*. When an excess carbon source such as glucose was supplemented into the medium, organic acids such as formic acid, acetic acid, and lactic acid could be generated due to the imbalance of the glycolytic pathway and TCA cycle. As a result, the pH of the medium usually dropped below 5.0, which severely inhibited the normal growth of *E. coli* used for microbial production. To overcome this inhibiting effect, an alkaline substance such as ammonium hydroxide, sodium hydroxide, sodium carbonate, or sodium bicarbonate should be added which increased the total cost of fermentation. Therefore, the development of an acid-resistant *E. coli* could save the cost of alkaline substances and facilitate the fermentation process with a relatively wide pH range.

Based on numerous acid-tolerant systems in *E. coli* MG1655, a set of acid-tolerance modules were constructed by fine-tuning the expression of the proton-depletion acid-tolerance system GadE, periplasmic chaperone HdeB, and reactive oxygen species scavenging systems [76]. In 2022, Yao et al. designed a set of synthetic acid-tolerance modules. Directed evolution was applied to construct an acid-responsive asr promoter library, from which four variants were selected and used in the synthetic modules of L-lysine. The best strain exhibited a similar lysine titer and yield at pH 6.0 comparable to the parent strain at pH 6.8 [76]. In addition, a recent study identified a novel strategy to increase D-lactic acid production based on the acid-tolerant system of *E. coli* by overexpressing the hydrogenase auxiliary proteins HypB and HypC, which resulted in a 336.3-fold increase in the survival rate of D-lactic acid-producing strains. In addition, D-lactic acid titers also exhibited a 113.6% increase [77]. Reconstruction of the *E. coli* membrane by enabling the synthesis of novel membrane component trans unsaturated fatty acids (TUFA) represent another strategy. As a result, tolerance to exogenously added octanoic acid and production of octanoic acid were both increased relative to the wild-type strain [78]. It was also found that overexpression of RffG, a key protein in the polyketide synthesis pathway, could effectively improve the survival rate of acid-tolerant strains under organic acid stress. Compared with the control group, *E. coli* overexpressing RffG showed a 4509.6-fold increase in survival at pH 4.0, which is the highest lactic acid-tolerance performance reported to date [67].

On the other hand, discharge of industrial wastewater exhibits a great threat to the environment and human health due to the harmful substances contained in wastewater, including heavy metals and organic substances, leading to serious pollution of water resources, soil, and the atmosphere. Chemical treatment is mainly employed for dealing with industrial wastewater, but this method will produce a large number of by-products, and the need to invest in a variety of acidic and alkaline chemicals which is costly. When confronted with acidic industrial wastewater, strains must be both tolerant to acid stress and capable of handling harmful substances. Bioremediation of heavy metals by acid-tolerant bacterial strains includes a variety of mechanisms, such as biosorption, bioaccumulation, biodegradation, bioassimilation, biotransformation, and bioprecipitation. Acid-tolerant strains can also degrade various hydrocarbons such as benzene, toluene, and xylene [79]. For example, wastewater from the mining industry is usually characterized by relatively high concentrations of soluble sulfate and metal ions, and the wastewater is usually acidic. Recently, a green and sustainable bioremediation strategy has been proposed to treat wastewater from the mining industry with microorganisms to remove metals and sulfates before discharge. The strategy involves immobilizing a mixed colony of cold-resistant sulfate-reducing bacteria (SRB) and other microorganisms on glass beads and treating the sulfate continuously in an upflow biofilm reactor. In the meantime, the protonaceous acidity of the wastewater is neutralized by microbial sulfation and the metals precipitate and settle out in the biofilm reactor [80]. Due to low bioavailability, the bioprecipitates have a low environmental risk [81]. Although there are still many harmful metals that cannot be removed, the engineering of wild strains for improving the resistance of acid and metals can solve this problem in subsequent studies. For instance, introducing phytochelatin synthase from the corn wine fission yeast SpPCS into *E. coli* could promote cadmium uptake effectively [82].

## 7. Conclusions

**Figure 3:**
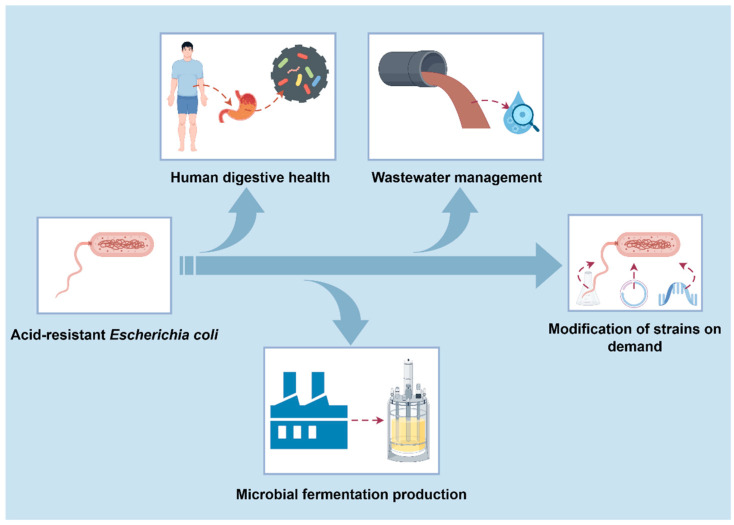
Acid-resistant *Escherichia coli* in industry.

Microorganisms are often subjected to acid stress during industrial fermentation, which inhibits bacterial growth and even affects product synthesis. In the face of acidic stress, *E. coli* has evolved a variety of acid-tolerant mechanisms to adapt to the acidic environment, including proton depletion, cell membrane protection, and macromolecular-repair mechanisms. Moreover, based on the acid-tolerance mechanism of *E. coli*, modified strains that can adapt to certain acidic environments can improve the production capacity of microorganisms in industrial fermentation (Figure 3). However, a comprehensive understanding of the acid-tolerance mechanism of *E. coli* should be further investigated, due to the intricate and collaborative metabolic network of *E. coli*. In addition, present regulatory mechanisms were not sufficient for developing an acid-tolerant *E. coli*. Through the combination of genetic engineering, metabolic engineering, and synthetic biology, great breakthroughs may be achieved in the area of acid-tolerant *E. coli* in the future. In order to better understand the mechanism of acid tolerance in *E. coli*, many issues should be addressed in the future. For example, the acid-tolerance mechanism of the acid-resistance AR system is still poorly understood; whether the acid-tolerance mechanism and acid-tolerance genes in other strains are also applicable to *E. coli*? When *E. coli* is applied in the industrial production of valuable chemicals, it may be exposed to a variety of stress conditions such as temperature, osmotic pressure, alkali, and so on due to the complex fermentation environment, and whether there is a synergistic or inhibitory relationship between them and acid stress needs to be investigated in the future.

On the other hand, more research is also needed for a more comprehensive understanding and summary of the acid-stress response in *E. coli*. As a narrative review, we do not intend to exhaust the subject regarding the acid-tolerance mechanism of *E. coli* or the industrial application of the acid-tolerance of *E. coli*, but to open the opportunity for discussing the development aspect in this field.
